# Identification of SSRI-evoked antidepressant sensory signals by decoding vagus nerve activity

**DOI:** 10.1038/s41598-021-00615-w

**Published:** 2021-10-26

**Authors:** Christine L. West, Karen-Anne McVey Neufeld, Yu-Kang Mao, Andrew M. Stanisz, Paul Forsythe, John Bienenstock, Denise Barbut, Michael Zasloff, Wolfgang A. Kunze

**Affiliations:** 1 Brain-Body Institute, St. Joseph’s Healthcare, Hamilton, ON Canada; 2 Department of Biology, McMaster University, Hamilton, ON Canada; 3 Department of Psychiatry and Behavioural Neurosciences, McMaster University, Hamilton, ON Canada; 4 Department of Pathology and Molecular Medicine, McMaster University, Hamilton, ON Canada; 5 Department of Medicine, McMaster University, Hamilton, ON Canada; 6 Enterin, Inc., Philadelphia, PA USA; 7 MedStar-Georgetown Transplant Institute, Georgetown University School of Medicine, Washington, DC USA; 8 Firestone Institute for Respiratory Health, St. Joseph’s Healthcare, Hamilton, ON Canada

**Keywords:** Neurophysiology, Physiology, Gastroenterology

## Abstract

The vagus nerve relays mood-altering signals originating in the gut lumen to the brain. In mice, an intact vagus is required to mediate the behavioural effects of both intraluminally applied selective serotonin reuptake inhibitors and a strain of *Lactobacillus* with antidepressant-like activity. Similarly, the prodepressant effect of lipopolysaccharide is vagus nerve dependent. Single vagal fibres are broadly tuned to respond by excitation to both anti- and prodepressant agents, but it remains unclear how neural responses encode behaviour-specific information. Here we demonstrate using ex vivo experiments that for single vagal fibres within the mesenteric neurovascular bundle supplying the mouse small intestine, a unique neural firing pattern code is common to both chemical and bacterial vagus-dependent antidepressant luminal stimuli. This code is qualitatively and statistically discernible from that evoked by lipopolysaccharide, a non-vagus-dependent antidepressant or control non-antidepressant *Lactobacillus* strain and are not affected by sex status. We found that all vagus dependent antidepressants evoked a decrease in mean spike interval, increase in spike burst duration, decrease in gap duration between bursts and increase in intra-burst spike intervals. Our results offer a novel neuronal electrical perspective as one explanation for mechanisms of action of gut-derived vagal dependent antidepressants. We expect that our ex vivo individual vagal fibre recording model will improve the design and operation of new, extant electroceutical vagal stimulation devices currently used to treat major depression. Furthermore, use of this vagal antidepressant code should provide a valuable screening tool for novel potential oral antidepressant candidates in preclinical animal models.

## Introduction

It is currently unknown whether oral serotonin reuptake inhibitor antidepressants (SSRIs) and mood-altering chemicals act directly on the brain or whether they act initially on the peripheral nervous system innervating the gut^1,2^. Peripherally, SSRIs might increase systemic levels of serotonin given that the gut contains the largest reserve of serotonin in the body^3^. Alternatively, they might act by activating serotonin-evoked gut to brain action potential transmission. Recent research^1^ has demonstrated that SSRIs exert their antidepressant actions through gut to brain signalling via the intact vagus nerve^4^. Consequently, vagal electrical signals that influence mood would be expected to have specific action potential firing characteristics.

Vagal chemosensing has long been recognised as being involved in behavioural and physiological regulation^5^. Such chemosensing may involve substances that translocate across the epithelial barrier to stimulate vagal endings, mediators released by epithelial enteroendocrine cells^6^ or transmitters released by intrinsic primary afferent neurons (IPANs) of the enteric nervous system (ENS)^7^. Irrespective of the nature and intramural location of chemosensory transduction, all vagal afferent signalling must be conveyed to the brain via nerve fibres as they leave the intestine within the mesenteric nerve bundle.

**Figure 1:**
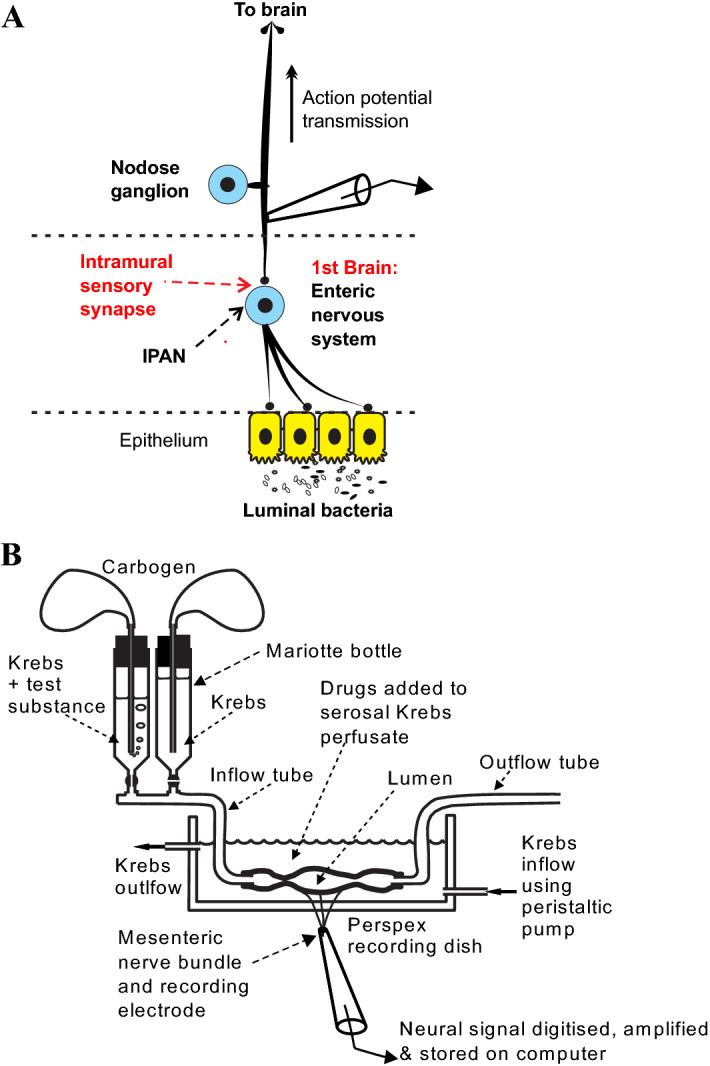
Gut to brain vagal afferent signals were recorded when the mesenteric nerve bundle emerges from the small intestine. (**A**) The enteric nervous system (ENS), which first appeared in cnidarian, developed communication pathways with the brain in Bilateria including rodents. Vagus-dependent intraluminal chemosensory signals that affect mood and affect-related behaviour are relayed to the visceral afferent vagus via intramural ENS sensory synapses. (**B**) Afferent multiunit extracellular action potentials were recorded via a suction electrode from a jejunal mesenteric nerve bundle attached to a 2–3 cm long jejunal segment placed within a modified Trendelenburg preparation.

Anterograde intra-axonal tracing studies demonstrate that the rodent ENS receives the densest and most abundant afferent innervation from the vagus nerve^8^ where vagal intraganglionic laminar endings (IGLEs) form intramural sensory synapses with IPANs^7^ (Fig. 1a).

Hypothetically, vagal mesenteric afferent fibres can encode sensory information using a labelled line code where each single afferent or a group of similarly tuned afferents is selective for one stimulus only; or an interval code whose firing pattern in a single fibre contains the information as a unique identifier for the nature of the peripheral stimulus. Individual chemosensory afferent fibres are thought to be broadly tuned (polymodal) group IV fibres responding to a variety of chemical and mechanical stimuli^8,9^. A recent comprehensive study^10^ using in vivo calcium imaging of rat vagal neurons has reported that chemosensory neurons were polymodal responding to a variety of intraluminally applied behaviourally active chemicals.

Varieties of pro- or antidepressant substances that are ingested or applied intra-abdominally are vagus-dependent for their behavioural effects to be evident. Intra-abdominal injection of the gram-negative *Escherichia coli* cell wall component lipopolysaccharide (LPS) has been shown to induce behavioural depression in rats which outlasted sickness behaviour and cytokine responses. However, prior subdiaphragmatic vagotomy abolished the depressive response although not LPS-induced circulating cytokines^2^.

Inbred BALB/c mice have elevated anxiety-like behaviour and are considered a reliably responsive strain in behavioural tests of depression^11^. We have previously reported^12^ that for this mouse strain, the antidepressant behavioural effects with the animals receiving chronic dosing (repeated doses via their drinking water) oral *Lactobacillus rhamnosus* JB-1 (JB-1), or of the selective serotonin receptor antagonists (SSRI) sertraline or fluoxetine, but not of the norepinephrine-dopamine receptor inhibitor bupropion, are abolished by prior subdiaphragmatic vagotomy^1^. Interestingly, subdiaphragmatic vagotomy in humans appears to be associated with increased psychiatric disorders^13^.

Application of intraluminal LPS^14^, a prodepressant stimulus or JB-1 (a psychoactive Lactobaccillus with antidepressant-like potential^12^), sertraline or fluoxetine^1^ activate vagal afferent fibres with an onset latency of 10–15 min. Here we aimed to determine whether individual vagal afferent fibres are broadly or specifically tuned; and if broadly tuned, individual afferents encode pro-or antidepressive signals in an action potential interval code. We addressed these questions using a validated ex vivo mouse jejunal segment perfusion preparation to record afferent single unit vagal activity using extracellular suction electrodes^1,15,16^ with luminal exposure to antidepressants or LPS (Fig. 1b). For the present paper and as previously described by Nullens et al.^17^ and Rong et al.^15^, extracellular action potentials (multiunit responses) were recorded from jejunal mesenteric nerve bundles, and single units representing extracellular discharge from individual single vagal fibres were discriminated by their unique spike waveform shape and amplitude. We identify the defining temporal characteristics of the "serotonergic antidepressant code". We analysed the afferent signals carried by single fibres of the mesenteric nerve following introduction of various anti- and pro depressant substances into the intestinal lumen. We show that vagus-dependent antidepressants share a common afferent neural firing pattern code that can be qualitatively and quantitatively distinguished from neural signals evoked by prodepressant agents.

## Results

### Pro- and antidepressants excite vagus

An average of 4 mesenteric nerve single units were responsive to CCK; for comparison Nullens et al.^17^ recorded between 4–5 vagal single units using an identical protocol for 9 eight-week-old male OF-1 mice. In contrast to reports for rats^18^, no single unit responded to serosal application of 0.1 mM of the 5-HT_3_ receptor agonist SR 57227. Individual vagal single units were broadly tuned responding with excitation to both pro- and antidepressant luminal stimuli.

**Figure 2:**
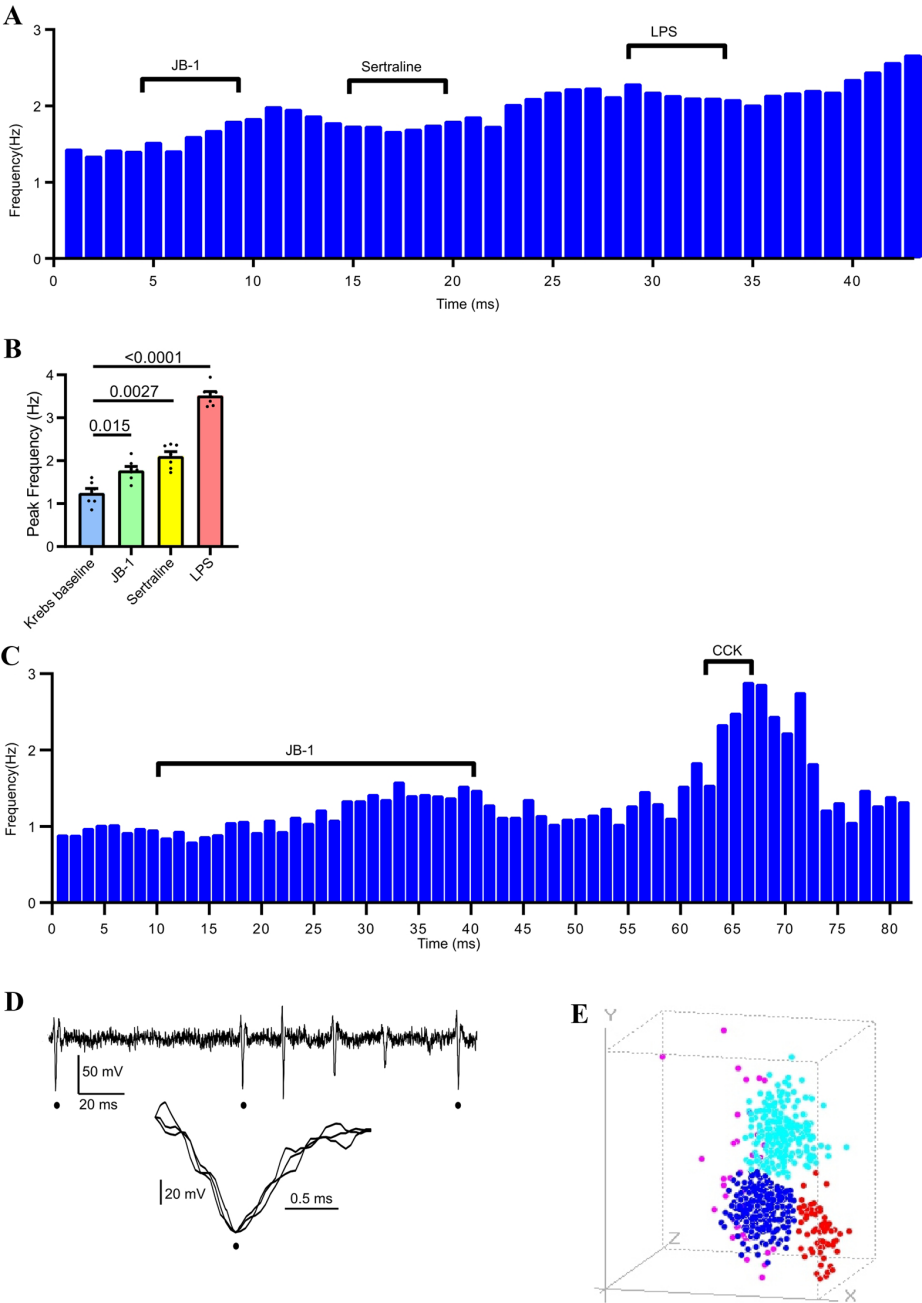
Single unit analysis of multimodal vagal mesenteric afferent responses to intraluminal chemical stimuli. (**A**) Histogram demonstrating polymodal responses to luminal stimuli for a vagus fibre single unit which was excited by 2 antidepressant agents (JB-1 or sertraline) and a pro-depressant agent (LPS). (**B**) both antidepressants and LPS increased vagal single unit firing rate (n = 7). Probabilities associated with no difference given by lines between bars (Holm–Sidak's multiple comparisons test). Data are mean ± s.e.m. (**C**) sequential rate histogram showing single unit response to 30 min intraluminal application of *Lactobacillus rhamnosus* JB-1 in a fibre that was identified as being a vagal afferent by its vigorous response to a brief application of 0.1 µM cholecystokinin (CCK). (**D**) Representative multiunit recording (from male BALB/c jejunal segments) showing identification of 3 single units with identical waveforms (peaks indicated by filled circles). Insert shows 3 units aligned at their peak. (**E**) Individual single units were identified according to their shapes using principal component analysis (PCA) algorithm within the Dataview programme. PCA resolved 4 single unit shape clusters for the parent recording. Three clusters (red, blue and aqua) were distinctly delimited, identifying 3 different single units uncontaminated by temporally coincident spikes of a different shape. The single dispersed cluster (magenta) represents spikes that were coincident so that their action potentials distort each other; these were not used for further analysis.

Brief (5 min) duration intraluminal application of the vagus-dependent antidepressant agents 1 × 10^9^ cfu JB-1 or sertraline excited the same single unit which was then further excited when 1 mM LPS was added to the lumen (Fig. 2A). The drugs were washed out between treatments by switching the intraluminal inflow tube to one containing only Krebs buffer. All single units tested by addition of these 3 agents were excited by the 3 substances, verifying that the individual vagal afferents were tuned to respond to both pro-and antidepressant classes of mood-altering intraluminal agents (Fig. 2B).

### Mean interspike interval effect sizes

**Figure 3:**
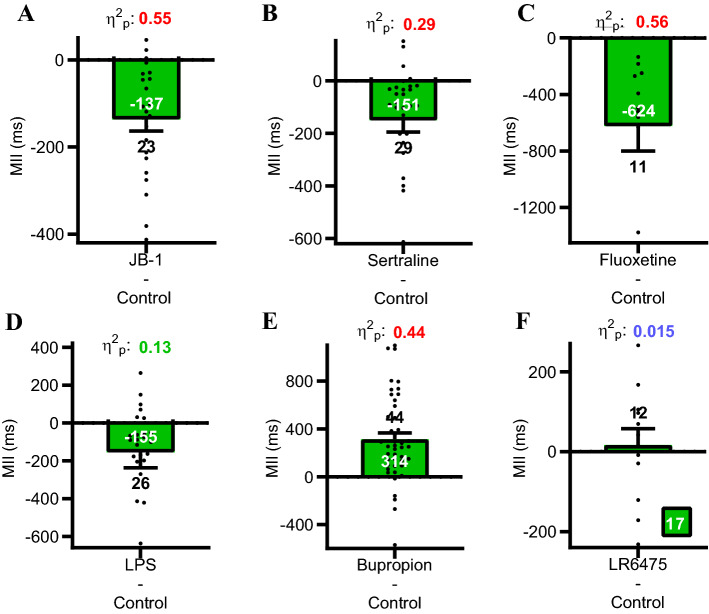
Paired differences graphs of mean interspike intervals before and after adding pro-, antidepressants or controls to the jejunal lumen. (**A**–**D**) vagus-dependent antidepressant agents (JB-1, sertraline and fluoxetine) and LPS decreased mean interspike intervals (MII) compared to paired Krebs control recordings. (**E**–**F**) Vagus-independent antidepressant bupropion increased MII and the non-antidepressant bacteria *Lactobacillus reuteri* ATCC PTA 6475 had little effect on MII. Effect sizes were calculated using the partial eta squared statistic η^2^p. Antidepressant agents produced a large effect, LPS a medium effect, and LR6475 had a small effect; see statistical analysis in “Methods”. Single unit numbers given above or below s.e.m, MII means given within solid bars.

**Figure 4:**
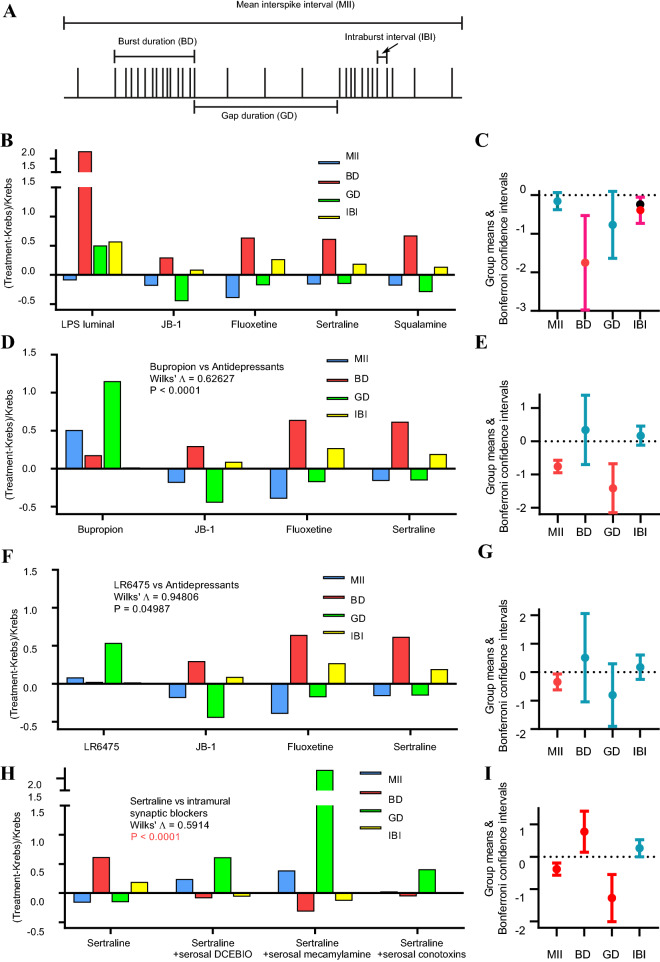
Vagus-dependent antidepressants had a unique spike firing pattern code that was disrupted by inhibition of IPAN to vagus synaptic transmission. (**A**) Diagram of stylised single unit discharge illustrating the 4 parameters measured to quantify firing patterns. (**B**) Fractional differences for the firing parameters showing a significant difference according to Wilk’s statistic for LPS versus vagus-dependent antidepressants and squalamine. Number of mice used were 6 for LPS (26 single units), 6 for JB-1 (23 single units), 3 for fluoxetine (11 single units), 7 for sertraline (29 single units), 3 for squalamine (12 single units), 10 for bupropion (44 single units) and 3 for LR6475 (12 single units). Panel (**C**) gives group (for all treatments) means and their combined Bonferroni confidence intervals. Intervals for BD and IBI did not straddle 0 indicating that both parameters in combination were responsible for the statistical difference given by Wilk’s statistic. (**D**) Significant difference between vagus-independent bupropion and vagus-dependent antidepressants. MII and GD in combination were responsible for this difference (**E**). (**F**) Non-antidepressant bacterium (LR6475) had significantly different firing parameters than the antidepressants. Only the MII parameter was responsible for generating this difference (**G**). (**H**) The intrinsic primary afferent neuron (IPAN) silencer DCEBIO (5 µM), the nicotinic receptor blocker mecamylamine (50 µM) or the ω-conotoxins GVIA and MVIIC (0.5 µM) disrupted the antidepressant code evoked by sertraline. No. of single units tested were 7 (2 mice) with DCEBIO, 6 (2 mice) with mecamylamine and 12 (3 mice) with conotoxins. I, MII, BD and GD together were responsible for the difference shown in (**H**). All mice were of the male BALB/c strain.

To quantify vagal single unit firing rates, we recorded mean interspike intervals (MII) in response to 30 min intraluminal applications of vagus-dependent antidepressants (JB-1, sertraline or fluoxetine), pro-depressant LPS, vagus-independent antidepressant bupropion^1^ or bacteria *Lactobacillus reuteri* 6475 used as a potential control. All vagus-dependent stimuli, JB-1, sertraline, fluoxetine, and LPS decreased MII. Bupropion, whose antidepressant effects do not depend on an intact vagus, increased MII. The anti-and pro-depressant agents elicited medium to large effect sizes in their paired (Krebs vs. intraluminal agent) differences (Fig. 3). Since vagus-dependent antidepressant agents and prodepressant agents all decreased MII in the same single fibre, the single unit firing code for the affective nature of the stimulus should be encoded in the 4 parameters (MII, gap duration (GD), burst duration (BD) and intraburst interval (IBI) that define the single-unit firing burst patterns (Fig. 4A).

### Acute antidepressant code

All of the vagus-dependent antidepressant agents as well as the vagus stimulating aminosterol squalamine^19^ evoked the same unique temporal pattern code that was statistically different from the code produced by the prodepressant LPS. We included squalamine in the code analysis because the vagus and myenteric IPAN stimulating actions of intraluminal squalamine have been suggested to have antidepressant behavioural effects (United States Patent Application 2018000237). The codes were calculated after determining the fractional difference (treatment-Krebs)/Krebs for each of the 4 firing parameters. The pametets measured were: mean interspike interval (MII), burst duration (BD), gap duration (GD) and intraburst interval (IBI) that define the single-unit firing burst patterns. Although all four antidepressant agents and squalamine decreased MII, LPS increased BD and IBI to a larger degree, and increased GD whereas the antidepressants all decreased GD (Fig. 4B). Group means and Bonferroni confidence intervals^20^ were used to determine which of the 4 parameters accounted for the observed differences. The combination of BD and IBI were responsible for the statistical difference between LPS and the vagus dependent antidepressants (Fig. 4C).

The code for the vagus-independent antidepressant bupropion was then compared to the vagus-dependent antidepressant agents. We predicted that vagal dependency would be essential if there were a cause-and-effect relationship between the antidepressant code and antidepressant behavioural changes. Indeed, our results significantly and statistically supported this prediction. Bupropion increased MII, BD, and GD but had a negligible effect (fractional difference of zero) on IBI (Fig. 4D). This code was also visibly different from that observed for LPS (cf. Fig. 4B). Bonferroni confidence intervals showed that a combination of MII and GD were responsible for the statistical difference between bupropion and other antidepressants (Fig. 4E).

**Figure 5:**
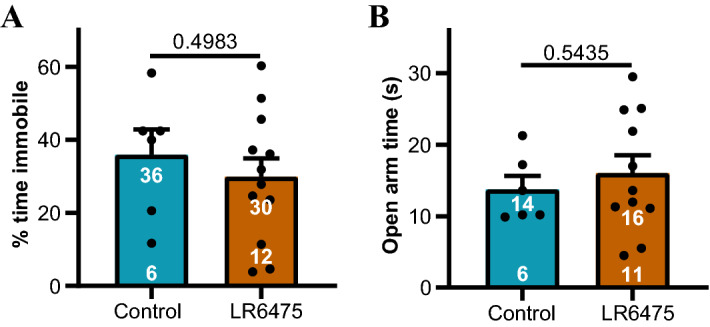
Ingestion of the probiotic LR6475 did not elicit antidepressant or antianxiety behavioural effects in BALB/c mice. (**A**) The percentage of time spent immobile in the tail suspension test for behavioural depression was not statistically different whether mice were fed water or water containing LR6475. (**B**) There was no statistical difference between the amount of time spent in the open arm of an elevated plus maze test for anxiety-related behaviour for animals fed water or LR 6475. Sample sizes are given at the bottom of filled bars and means are at the top of the bars. Data are means ± s.e.m. Probabilities under the null hypothesis of no difference given above parallel lines according to unpaired t-tests.

We used probiotic *Lactobacillus reuteri* 6475 (LR6475) as a control to test whether antidepressant activity is generically inherent in *Lactobacillus* probiotics. We chose LR6475^21^ since its potential effects on mood or related behaviours are currently unspecified. LR6475 evoked a firing code different from that for vagus-dependent antidepressants and increased MII and GD fractional differences with minimal effects on BD and IBI (Fig. 4F). Bonferroni confidence intervals showed that MII only was responsible for this statistical difference (Fig. 4G). Since LR6475 did not evoke the antidepressant firing code, we tested the predictive power of our acute code analysis method (as described in the “Analysis of single unit firing patterns” Methods section) on the effects of ingestion of this bacterium on antidepressant behaviour (tail suspension test) and antianxiety behaviour (elevated plus maze). As opposed to the effects of JB-1, chronic (15 days) ingestion of LR6475 had no statistically significant effect on antidepressant or antianxiety behaviours in BALB/c mice (Fig. 5).

The antidepressant code depended on intramural synaptic transmission from IPANs to the afferent vagus. Greater than 67% of the vagal afferent single unit response to intraluminal JB-1^7^ requires synaptic transmission between myenteric IPANs and abutting vagus afferent fibres via a nicotinic intramural sensory synapse between IGLEs and IPAN somata^7^ (Fig. 1A). For BALB/c mice, SSRI antidepressants such as sertraline excited vagal afferents and myenteric IPANs and are dependent on an intact vagus for their antidepressant behavioural effects^1^. Therefore, we tested if intramural synaptic transmission from IPANs to IGLEs is necessary for the generation of the antidepressant code evoked by intraluminal application of sertraline. This pathway was interrupted by inhibiting IPAN action potential firing with the intermediate conductance calcium-dependent K^+^ (IK_Ca_) channel opener DCEBIO, blocking synaptic transmission using the Ca^2+^ ion channel blockers ω-conotoxins GVIA and MVIIC, or by blocking the nicotinic acetylcholine receptor (nAchR) with the nAchR antagonist mecamylamine^7^. We added the inhibitors or each of the blockers to the gut serosa in separate experiments prior to luminal perfusion with sertraline. Addition of DCEBIO, mecamylamine, or the ω-conotoxins disrupted the vagal firing code produced by sertraline in their absence (Fig. 4H). According to Bonferroni confidence intervals, this statistical difference could be attributed to alterations in MII, BD, and GD, but not IBI (Fig. 4I).

### Feeding SSRIs yields antidepressant code

**Figure 6:**
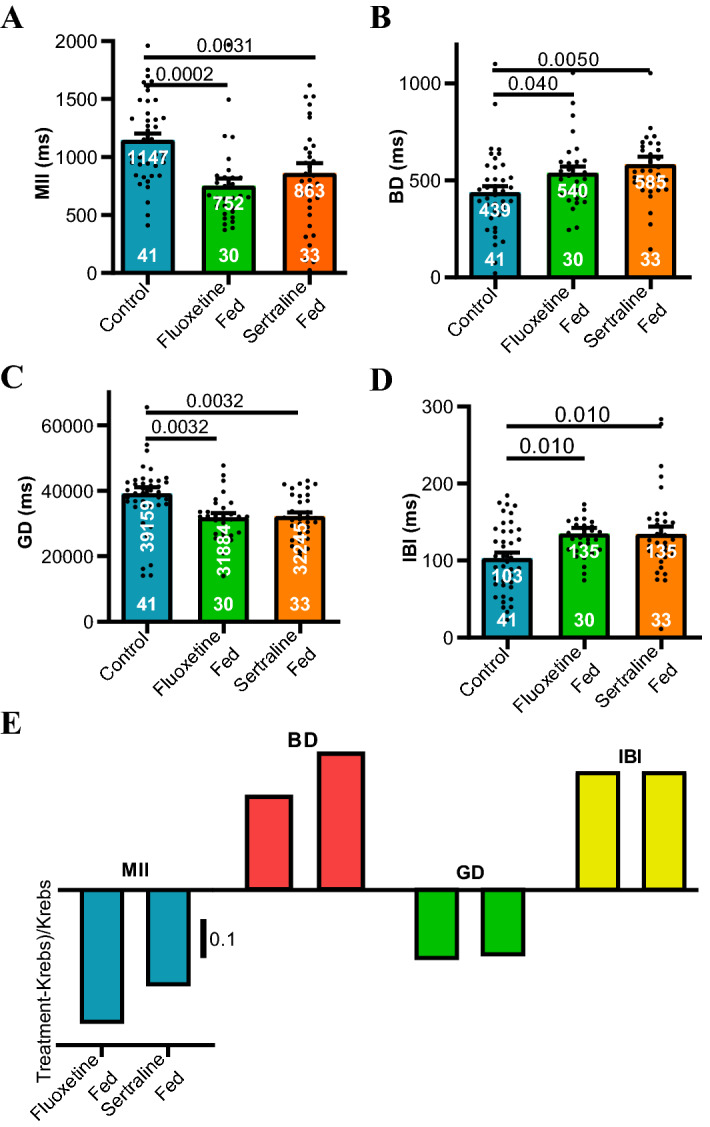
Prior feeding of sertraline or fluoxetine recapitulates the effects of their acute intraluminal application. 14-day feeding with either antidepressant (**A**) reduced, (**B**) increased, (**C**) reduced and (**D**) increased MII, BD, GD, and IBI respectively. Control animals were fed water only (10 mice), while fluoxetine fed (7 mice received 18 mg/kg added to water and sertraline fed (8 mice) received 6 mg/kg daily for 14 days. Animals were sacrificed on the 15th day for ex vivo measurements resting afferent vagal discharge. Probabilities under the null hypothesis of no difference given above parallel lines according to Holm–Sidak's multiple comparisons tests. Sample sizes are given at the bottom of filled bars and means are at the top of the bars. Data are means ± s.e.m. (**E**) When the means from (**A**–**D**) were plotted as fractional differences [(treatment − Krebs)/Krebs], codes comparable to those observed after acute administration of the antidepressant drugs were evident (compare Fig. 4B).

We tested whether oral feeding of the SSRIs sertraline or fluoxetine repeated the firing code evoked by acute ex vivo SSRI exposure. Each of the four firing parameters were measured ex vivo after BALB/c mice were fed for 14 days with either sertraline, fluoxetine in water or plain drinking water^1^. Animals were sacrificed on day 15 and firing parameters of single unit vagal fibres measured ex vivo with only Krebs buffer perfusing the lumen. Both fluoxetine and sertraline notably decreased MII compared to mice who received only water (Fig. 6A). Fluoxetine and sertraline increased BD compared to the control (Fig. 6B). The SSRIs decreased GD compared to the control (Fig. 6C). Lastly, they increased IBI (Fig. 6D). The fractional differences for each of the firing patterns for fed mice calculated by subtracting each of the firing parameters after feeding from the average of the control parameters produced an antidepressant code comparable to that observed after acute SSRI administration (Fig. 6E).

### SW mice also show the antidepressant code

**Figure 7:**
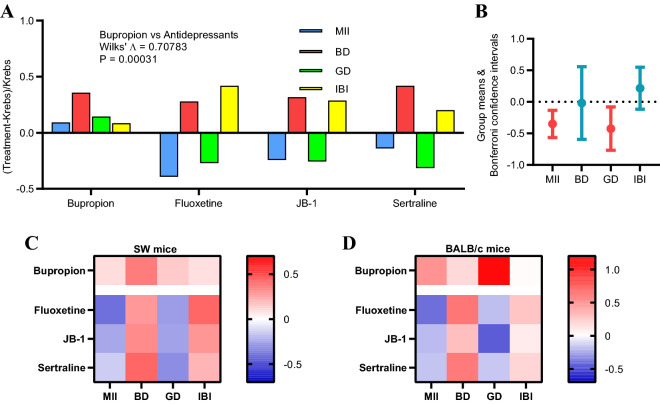
The vagal firing pattern code for antidepressants in SW mice is comparable to that for BALB/c mice. (**A**) Effects of adding vagus-independent (bupropion) and vagus-dependent antidepressants in acute before and after experiments for jejunal segments taken from male Swiss Webster (SW) mice. There was a significant difference (Wilk’s Λ) in fractional differences for the 4 firing parameters between bupropion and the other antidepressants. (**B**) As was the case for BALB/c mice, MII and GD in combination were responsible for this difference. (**C**,**D**) Heat maps of the fractional differences illustrating the essential similarity of the antidepressant codes for SW and BALB/c mice.

Mesenteric afferent nerve recordings were repeated with sertraline or fluoxetine in the Swiss Webster (SW) mouse strain to show that our findings were not strain specific. The vagus-dependent antidepressant agents or bupropion were tested intraluminally in ex vivo jejunal segments at the same concentrations used for BALB/c. Fluoxetine, JB-1, and sertraline all decreased MII, increased BD, decreased GD, and increased IBI in SW mice. This was significantly different from the pattern of firing produced by bupropion, which evoked an increase in all 4 firing parameters (Fig. 7A). Bonferroni confidence intervals identified MII and GD as the parameters responsible for this difference (Fig. 7B). Fractional differences for the firing parameters (x-axis) for each of the four treatment agents (y-axis) were contrasted for SW mice (Fig. 7C) vs. BALB/c mice (Fig. 7D) using heat maps. Blue represents a negative fractional change, while red represents a positive fractional change. The heat maps illustrate that the antidepressant firing codes were qualitatively similar between SW and BALB/c mice.

### Antidepressant code is conserved across sexes

**Figure 8:**
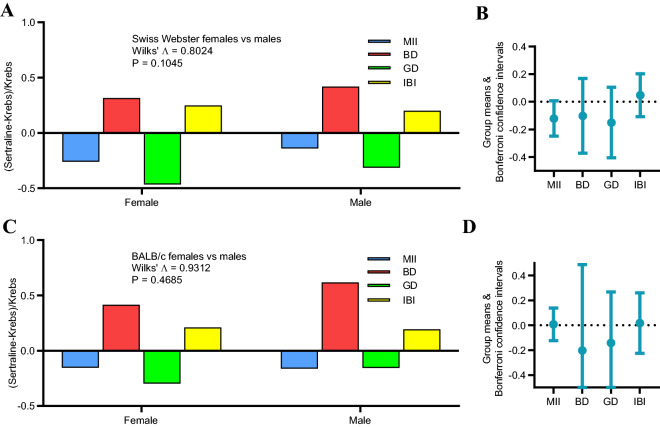
Conservation of antidepressant code between sexes. (**A**) Both 4 male (N = 15 single units) and 6 female SW (N = 24 single units) mice revealed a similar pattern of fractional differences in code parameters with no statistically discernible difference in contrasts between sexes. (**B**) Bonferroni confidence intervals for all code parameters encompassed 0 confirming that there were no statistical differences between sexes. (**C**) A similar pattern in code parameters was also revealed for 7 male (N = 29 single units) and 6 females BALB/c (N = 25 single units). (**D**) Bonferroni confidence intervals also spanned 0 for BALB/c mice.

The vagal code evoked by acute intraluminal sertraline was qualitatively similar for female compared to male SW mice. Both sexes revealed the canonical antidepressant code of decreased MII, increased BD, decreased GD, and increased IBI with no statistically discernible difference between sexes (Fig. 8A). A similar pattern was revealed for female and male BALB/c mice (Fig. 8C). Group means and Bonferroni confidence intervals for each of the 4 parameters confirmed the lack of sex differences (Fig. 8B,D).

## Discussion

We have used ex vivo afferent vagal single unit analysis of multiunit recordings taken from the mouse mesenteric nerve bundle as it emerges from a segment of the proximal jejunum. We confirmed that both pro- and antidepressant vagus-dependent agents applied intraluminally excite the same single unit, whereas a vagus-independent antidepressant or the non-antidepressant bacteria LR6475 had no excitatory effects. Antidepressant and prodepressant vagus-dependent agents evoked distinctive antidepressant and prodepressant codes respectively. Pharmacological silencing of intramural IPAN to afferent vagus neurotransmission abolished vagal firing and the antidepressant code induced by acute application of sertraline. The antidepressant code was preserved even when SSRI antidepressants were previously fed rather than acutely administered possibly because repeated stimulation of myenteric neurons by the drugs could lead to long lasting persistent increases in the intrinsic excitability of these neurons^22^.

Others^10^ have also reported that individual rodent afferent vagal fibres identified by their excitatory response to CCK are polymodal being excited by intraluminal amino acids, fatty acids or glucose, although anti-and prodepressant agents were not tested. Zanos et al.^23^ recorded multiunit signals from the mouse cervical vagus after IP injection of either TNF or IL-1β and, similar to our approach (see “Methods”), identified single units by principal component analysis of spike shapes. They concluded that the 2 cytokines activated different populations of afferent vagal single units (labelled line code) and that each cytokine produced a unique firing pattern for which firing rate was the main discriminating variable. The labelled line code is also evident in other peripheral chemoceptive afferents such as taste receptors^24^.

Single doses of SSRIs have previously been shown to have behavioural antidepressant-like effects in murine models. To the best of our knowledge these have been performed using intraperitoneal injections of a SSRI, see for example Medrihan et al.^25^. We have previously reported^1^ that a single intraperitoneal injection of either fluoxetine or sertraline had antidepressant-like effects in mice, but this effect was vagus-independent since the antidepressant action was not reduced by prior subdiaphragmatic vagotomy. No coded pattern of vagal pulses originating within the gut are likely to reach the brain after the vagotomy.

Unfortunately, we were unable to find an example in the literature where a single oral dose of an SSRI had an antidepressant-like behavioural effect in a mouse model using either tail suspension or forced swim tests. For our present paper, feeding SSRIs in the drinking water for 14 days did produce the code, but it is not possible to determine in this case how many individual doses were ingested per day per animal.

In other mouse model experiments, oral ingestion of SSRI antidepressants (see Cryan et al.^26^ for review) or vagus nerve stimulation^27,28^, repeated single doses were administered over periods of days. For the only experiment, cited in the literature, in which a single oral SSRI dose (fluoxetine) up to a maximal concentration of 30 mg/kg was given to mice, no statistical decrease in tail suspension test immobilisation time was reported^29^.

As is the case with VNS^27^, there is no a priori reason to suppose that a single acute activation of vagal nerves by intraluminally applied antidepressants should elicit a significant antidepressant-like behavioural response. It is more consistent with the literature^26^ on the use of antidepressants in animal models that repeated applications of the antidepressant would be required. Nevertheless, the consistency of the antidepressant code as it applies to SSRIs and the psychoactive JB-1 indicates that we have revealed a canonical antidepressant code for the sensory vagus nerve; which, like VNS, would need to be repeated for a statistically discernible behavioural effect to be evident.

The primary role of myenteric IPANs in relaying chemical signals from the luminal epithelium to vagal fibres as revealed in the present paper is consistent with the chemical coding of sensory neurites supplying the epithelium. Demonstrably, the densest innervation of intestinal epithelial layer cells is supplied by the ENS which provides more than 90% of sensory neuropeptide containing fibres innervating the mucosal layer^30,31^. Our results are also consistent with the report that the majority (> 2/3) of vagal afferents innervating the small intestine act as interneurons receiving synaptic signals from an intramural nicotinic sensory synapse^7^.

Squalamine also produced an antidepressant code comparable to that evoked by sertraline providing additional experimental support for a potential antidepressant role of this shark-derived aminosterol as proposed in the US Patent Application 2018000237. We predict that other vagus-dependent antidepressants, not yet tested in our preparation would, if applied intraluminally, generate a similar code to those tested for in the present paper since a signal is carried to the brain by a common afferent vagal pathway. This would be so irrespective of the different transduction mechanisms that might be involved for the various types of antidepressants at the epithelium lining the gut lumen or of changes in the resident microbiome since afferent vagal fibres represent the final common peripheral signalling pathway.

In addition, pathological alterations in myenteric IPAN function such as occur for example in intestinal inflammation^32^ could change constitutive or antidepressant-evoked afferent vagal firing potentially altering mood or prompting depressive behaviour. This interpretation could be important because the probiotic bacterium LR6475 which has no published antidepressant effects and does not generate the antidepressant vagal code is known to increase TNF production in human cells^21^.

In conclusion, our experiments suggest the existence of a canonical antidepressant code for afferent vagal signalling whose application in the context of therapeutic antidepressant vagal stimulation could improve the effectiveness of extant stimulation protocols which have been devised in the absence of such knowledge. Also, our ex vivo preparation could be used to screen other chemicals or bacteria for presence of an antidepressant code and thus potential antidepressant behavioural effects. Further research should investigate whether the code or a similar one applies to other mammals including humans^33^.

## Methods

Six- to eight-week-old male or female BALB/c (average weight 26 g) and male or female Swiss Webster (SW) (average weight 33 g) mice were purchased from Charles River (Montreal, QC, Canada) and allowed to habituate to the animal facility for 1 week. Animals were housed 4/cage and under controlled conditions (21 °C) on a 12-h light/dark cycle (lights on at 5:00 a.m.) and fed ad libitum. All experiments were carried out in accordance with the guidelines of the Canadian Council on Animal Care and ARRIVE Guidelines and were approved by McMaster University’s Animal Research Ethics Board (Animal Utilisation Protocols: 16-08-30 & 20-05-21).

Mice were killed by cervical dislocation and all action potential recordings performed ex vivo. Segments of excised proximal jejunum (2.5 cm) with attached mesenteric arcade containing a neuromuscular bundle were removed from freshly killed animals and immediately placed in a Sylgard 170 silicone elastomer (Dow Corning, Midland, MI, USA)-coated recording petri dish filled with Krebs buffer (in mM): 118 NaCl, 4.8 KCl, 25 NaHCO_3_, 1.0 NaH_2_PO_4_, 1.2 MgSO_4_, 11.1 glucose, and 2.5 CaCl_2_ bubbled with carbogen (95% O_2_–5% CO_2_). The segment was emptied of contents using a syringe filled with Krebs, then both oral and anal ends were cannulated with silicone tubing. The gut and mesenteric tissue were pinned to the Sylgard and the mesenteric nerve bundle exposed by microdissection under a stereomicroscope. The preparation was then transferred to an inverted microscope and the lumen gravity perfused at 1 ml/min with room temperature (22 °C) carbogenated Krebs or Krebs plus one of the luminal additives using several Mariotte bottles^34^ attached to a plastic manifold. The serosal compartment was separately perfused at 5 ml/min with prewarmed (34 °C) Krebs solution to which 3 µM nicardipine had been added to isolate vagal chemosensory responses by preventing active muscle contractions but not vagal responses to distension^16^.

The cleaned nerve was sucked into a glass-recording pipette attached to a patch-clamp electrode holder, and extracellular nerve recordings (Fig. 1A,B) made running pClamp software using a Multi-Clamp 700B amplifier and Digidata 1440A signal converter (Molecular Devices). The nerve bundle within the pipette was isolated from the Krebs within the recording dish by gently pressing the tip into fat tissue adherent to the uncleaned parts mesenteric arcade. Electrical signals were bandpass-filtered at 0.1–2 kHz, sampled at 20 kHz, and displayed and stored on a personal computer^16^.

Baseline recording with Krebs buffer in the gut lumen was performed for 15 min, after which the luminal perfusate was switched for 40 min to one containing Krebs buffer with one of the test substances added. Then the perfusate was again switched to Krebs and recording continued for 30 min. Rundown of constitutive vagal discharge in this system is not evident until > 90 min of recording^16^. Only one luminal test additive was applied once per animal to avoid possible signal rundown. Finally, we distended the intestine by raising the intraluminal pressure to 14 hPa to demonstrate that the isolated single units could still respond and were not subject to rundown. Subdiaphragmatic vagotomy abolishes all mesenteric nerve responses to CCK^16^ and testing for the response of each of the isolated single units to CCK is a well-established method for identifying vagal fibres within the mesenteric nerve bundle^16,35^. Cholecystokinin (25–33) sulphated (AnaSpec, Fremont, CA, USA) was dissolved in dimethyl sulfoxide (DMSO) to make a 1 mM stock solution. Aliquots were diluted on the day of the experiment to a working concentration of 0.1 µM in Krebs buffer, with a final DMSO concentration ≤ 0.0001%.

We tested the following “psychoactive” (change brain function or mood) agents: luminal administration of 10^9^ cfu/mL live *L. rhamnosus* (JB-1), 10 µM sertraline hydrochloride (MilliporeSigma, Burlington, MA, USA) 30 µM fluoxetine hydrochloride (MilliporeSigma), 10 µM bupropion hydrochloride (MilliporeSigma)^1^, or 1 mM LPS dissolved in pyrogen-free saline (*Escherichia coli* O127:B8, (MilliporeSigma)^2^. Squalamine dilactate was provided by Dr Michael Zasloff, Georgetown University (Washington, DC, United States). Squalamine dilactate powder was dissolved in 90% ethanol to make a stock solution, then aliquoted and stored at − 20 °C until use. Stock solution was diluted in Krebs buffer to a working concentration of 30 µM for in vitro experiments. Pilot experiments showed that these concentrations activate vagal fibres by at least 20% above baseline firing rates. In determining these concentrations, we acknowledge the difficulty in correlating drug or probiotic concentrations for in vivo vs ex vivo settings. We have found no cogent way to deduce the concentrations of these agents within parts of the in vivo ileum after an oral dose. It is for this reason that we had performed pilot experiments to determine the concentrations that increased the firing rates by at least 20% from those measured during Krebs only administration to the lumen. We found that lower concentrations evoking a smaller increase in firing rates made it more difficult to discern the onset of the excitatory responses in the action potential event traces within the Dataview analysis program.

### Analysis of single unit firing patterns

Each single unit type was identified as belonging to an individual vagal fibre by an excitatory response (increased firing rate) to applying cholecystokinin (CCK) to the serosal compartment of the preparation^18^, see Fig. 2C for a sequential rate histogram with a representatFive example. Single units were discriminated using principal component analysis based on their action potential shape, amplitude and width (Fig. 2D) using a dedicated program (Dataview^36^) for extracellular action potential analysis. Delimited clusters (Fig. 2E) of units marked by a single colour represented different single unit types. Single units belonging to each tight cluster were converted into a single event point processes and displayed as sequential rate histograms and used for further analysis.

The single unit events were partitioned into separate colour-coded point process event channels with each coloured channel representing a different unit type. Each coloured channel was further subdivided into (Krebs) and treatment (intraluminal agent) periods. For each event channel in Dataview the point processes were displayed as probability density functions (√Y) vs log(time) using the “event parameter histogram” plot option. A single exponential fit to this plot yielded the mean interspike interval (MII).

Event bursts were detected by the Poisson surprise method^37^, with a search value of 2, for which the surprise is defined as − log10(p), where p is the probability of a set of events occurring this close together by chance. Thus, a surprise value of 2 reflects a p value of 0.01. For each control or treatment event channel being measured in Dataview the “Event analyse: Histograms/statistics” option of the programme calculates the gap (GD) and burst (BD) durations.

For the intraburst intervals (IBI) and for each different single unit point process, the Krebs and treatment bursts event channels that were created by the Poisson surprise method were logically combined using the AND gate function, thus extracting only the bursts from the point process events. Then, selecting the burst channel for a given single unit as the gate channel and choosing the “Event analyse: Histogram/statistics” option, the IBI was extracted for either the control or treatment channels.

### Statistical analysis

The partial eta squared statistic *η*^2^_p_^38^, pp. 70–71 gave effect sizes for paired differences calculated in the t-test module within GraphPad Prism ver. 8.3 GraphPad Software, San Diego, USA. According to Cohen’s guidelines^39^ for interpreting *η*^2^_p_, 0.01 indicates a small, 0.06 a medium and 0.14 a large effect size.

For each identified single unit and for control or treatment conditions, MII, BD, GD and IBI (Fig. 4A) were pasted into an Excel 2016 spreadsheet and converted into fractional differences (Treatment − Krebs)/Krebs. Then single factor multivariate analysis was performed for each of the luminal agents using the Real Statistics Resource Pack software (Release 7.2. Copyright (2013–2020) Charles Zaiontz. www.real-statistics.com). Multivariate calculations provided the Wilk’s Lambda test statistics for contrasts between a single luminal agent and several others (see Fig. 4 for examples). Not all the 4 parameters may have encoded information about the difference between the different luminal agents. Therefore, we calculated the group means and Bonferroni confidence intervals for each of the 4 parameters. The Bonferroni confidence interval is equal to the (mean − standard error)/critical t-value for 0.05 significance for the lower bound, and (mean + standard error)/critical t-value for the upper bound. Only those parameters whose confidence intervals that did not span 0 contributed to the statistical differences between the luminal agents (see Fig. 4).
